# Generating Community Engaged Learning in Gerontology Courses During the COVID-19 Pandemic

**DOI:** 10.1093/geroni/igab046.293

**Published:** 2021-12-17

**Authors:** Katarina Felsted, Samantha Whitehead

**Affiliations:** University of Utah, Salt Lake City, Utah, United States

## Abstract

This presentation describes the core traits of a community-engaged learning (CEL course), how one gerontology program incorporated a theoretical framework to continue to provide students opportunities during the COVID-19 pandemic, and how generalizable this is across gerontology programs. Caregiving and Aging Families, a gerontology course enrolling both undergraduate and graduate students, champions community-engaged learning in two critical ways: students attend caregiver support groups in the community, and students form a partnership with a caregiver mentor in the community. This partnership allows students an intimate look at the caregiver's role and burden while enlisting the student to prepare a service care plan and compendium of resources for the caregiver. Ensuring the safety of older adults during the COVID-19 pandemic placed restrictive parameters on these experiences. While students typically attend support groups and identify and partner with a caregiver mentor in person, this needed modification during the pandemic. This was created through the application of Baltes' Theory of Selection, Optimization, and Compensation (SOC model), aided by a CEL teaching assistant, funded through the campus Community Service Center. This allowed for identifying, coordinating, and communicating with community partners throughout the semester and provided ongoing communication, technical assistance, and problem-solving for both partners and students. Caregiver groups with a robust online, synchronous presence were identified and approached. The gerontology program communities of interest disseminated a call for community caregivers with basic technological familiarity. The caregiver mentor-student partnerships were founded and maintained, with additional benefits stemming from a shared pandemic experience.

